# Construction of a virtual simulation laboratory for gene detection

**DOI:** 10.1186/s12909-023-04401-2

**Published:** 2023-06-08

**Authors:** Lin Yu, Wenjun Wang, Zhongmin Liu, Ze Liu, Yunjian Xu, Yongping Lin

**Affiliations:** 1 The First Affiliated Hospital of Guangzhou Medical University, Guangzhou, 510120 China; 2 KingMed School of Laboratory Medicine, Guangzhou Medical University, Guangzhou, China

**Keywords:** Gene research, Virtual reality technology, Experimental teaching of medical students

## Abstract

**Objective:**

The current paper aims to discuss the development of a virtual simulation experiment teaching system and review its effectiveness in improving the teaching of clinical skills to college medical students.

**Methods:**

Collaborators used 3D Studio Max, Unity 3D and Visual Studio to develop four modules: laboratory thinking training, biosafety training, gene testing and experimental assessment. Teaching was conducted and a virtual software program was used for evaluation of the students.

**Results:**

The laboratory safety training system, virtual gene experiment system and experimental assessment system were developed. The results of the questionnaire survey show that the software provides good interactivity and guidance. The interest of medical students in study is improved and they received training in clinical experimental thinking. Student evaluation assists their scientific research practice, and can improve the awareness of biosafety.

**Conclusion:**

The virtual simulation experiment teaching system, when applied in the teaching of undergraduate and postgraduate experiment courses, can bring about rapid improvements in the following areas: biosafety awareness, interest in learning about experiments and experimental skills, clinical experimental thinking, and comprehensive experimental ability.

## Introduction

The traditional process of cultivating medical students' experimental skills has the following characteristics: long experimental period, complex process, most of the experimental equipment is imported and expensive, the cost of experimental reagents is high, and the experimental workers are often not familiar with the operation of the instrument, resulting in process error, resulting in the waste of human and material resources. There are many risk factors in medical experiments. Careless operation will cause danger to human body, instruments and experimental sites.

At the same time, many freshman medical students lack experimental operation skills training or rigorous preparation for scientific research. Most of these students began experimental work under the guidance of senior students, often resulting in noncompliance with standard experimental procedures. If medical students do not undergo standardized training, the quality of their professional preparation will likely be compromised.

Previous studies have shown that virtual technology has many advantages in education, including improving learning efficiency and quality by feedback signals to the brain, sufficient free training time, accurate and automatic training data, etc. [1]. In China, in 2014, the Ministry of Education announced the first batch of national virtual simulation experiment centers; in 2018, the Ministry of Education selected the first batch of national virtual simulation experiment teaching projects; and in 2021, the Ministry of Education launched the development of a national virtual simulation first-class curriculum. The use of virtual simulation experiment courses in the teaching of medical students is increasing [2, 3], but there are few reports of virtual simulation experiment projects aimed at the biosecurity and scientific research innovation needs of medical students. Especially in the high-level biosafety laboratory,medical students need to understand the laboratory biosecurity measures before entering the laboratory. Virtual simulation experiments can allow students to learn to do different experiments, access laboratories of different biosafety levels, and get an understanding of when to use different types of protective apparel. Through the realistic simulation system, students can obtain real and profound memories without having to worry about their personal safety [4, 5].

Based on the above mentioned difficulties in medical education [6, 7], we urgently need to develop a virtual simulation experiment training system, so that medical students all over the country can have free access to learning resources at any time.

Therefore, based on the previous virtual simulation experiment project, this project aims to develop a virtual simulation experiment teaching system for medical students, including comprehensive training of biosafety protection and gene research. It enables medical students, especially during the COVID-19 epidemic, to learn to use the virtual simulation experiment teaching system anytime and anywhere, which is conducive to their success in subsequent real experiments, improving the success rate of experiments, and cultivating comprehensive experimental thinking. Since medical students need to have the ability to conduct preliminary scientific research and master various high-tech machinery and instruments, virtual simulation experiment teaching is crucial for them to carry out innovative scientific research and training [8].

## Contents and methods

### Contents

The first part of the simulation involves the biosafety of the laboratory, which all medical students need to understand before entering the laboratory. This study involves the development of biosafety protection module, mainly including wearing laboratory protective clothing and seven-step handwashing technique training, including the basic biosafety knowledge that medical students must be familiar with before conducting experiments.

The second part of the virtual simulation deal with common basic experiments used by medical students (such as genetic research and the operation of a PCR machine). It trains the students’ clinical experimental thinking before the experiment, so that students can have a clear understanding of the whole experiment before starting the experiment, and can independently design experiments according to different experimental objectives. These experiments are time-consuming, complicated and involve many reagents. Therefore, using virtual software for training can help students get familiar with and master the operation, principle and precautions of the experiment process before the actual experiments [9]. At present, most of the experiments carried out by medical students will involve genetics, so the basic experiments required for gene research developed in this study include comprehensive virtual interactive experiments in three aspects: gene extraction, gene amplification and gene testing. In the third part of the simulation, medical students enter the assessment mode after learning the system [10–12].

### Main research methods

#### Construction of biosafety protection training system

**Fig. 1:**
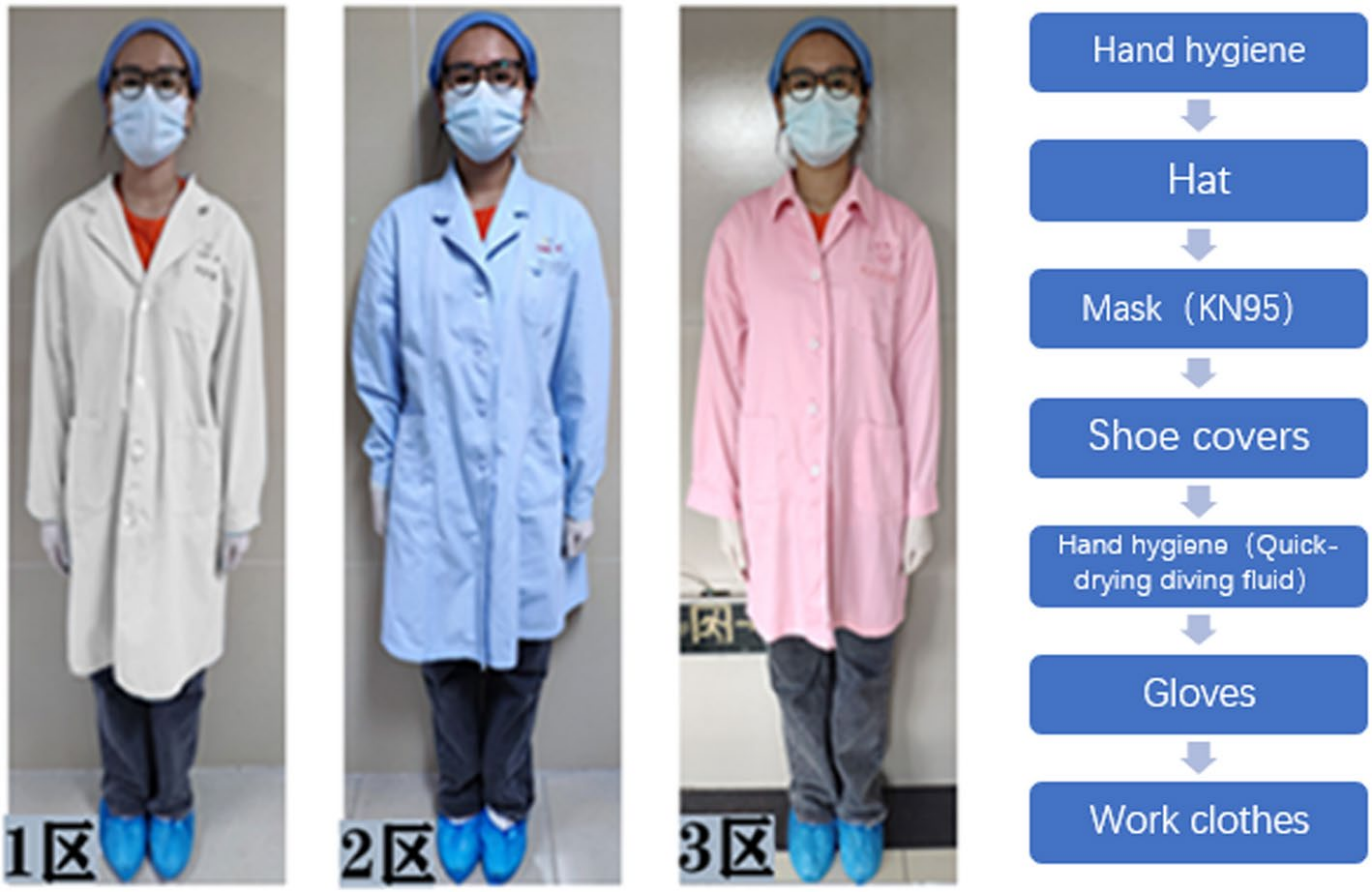
Personal protective equipment requirements for different parts of the PCR laboratory. Note: In the PCR laboratory, work clothes are distinguished according to different studios with different colors, Zone 1 (white) – Zone 2 (blue) – Zones 3 and 4 (pink)

**Fig. 2:**
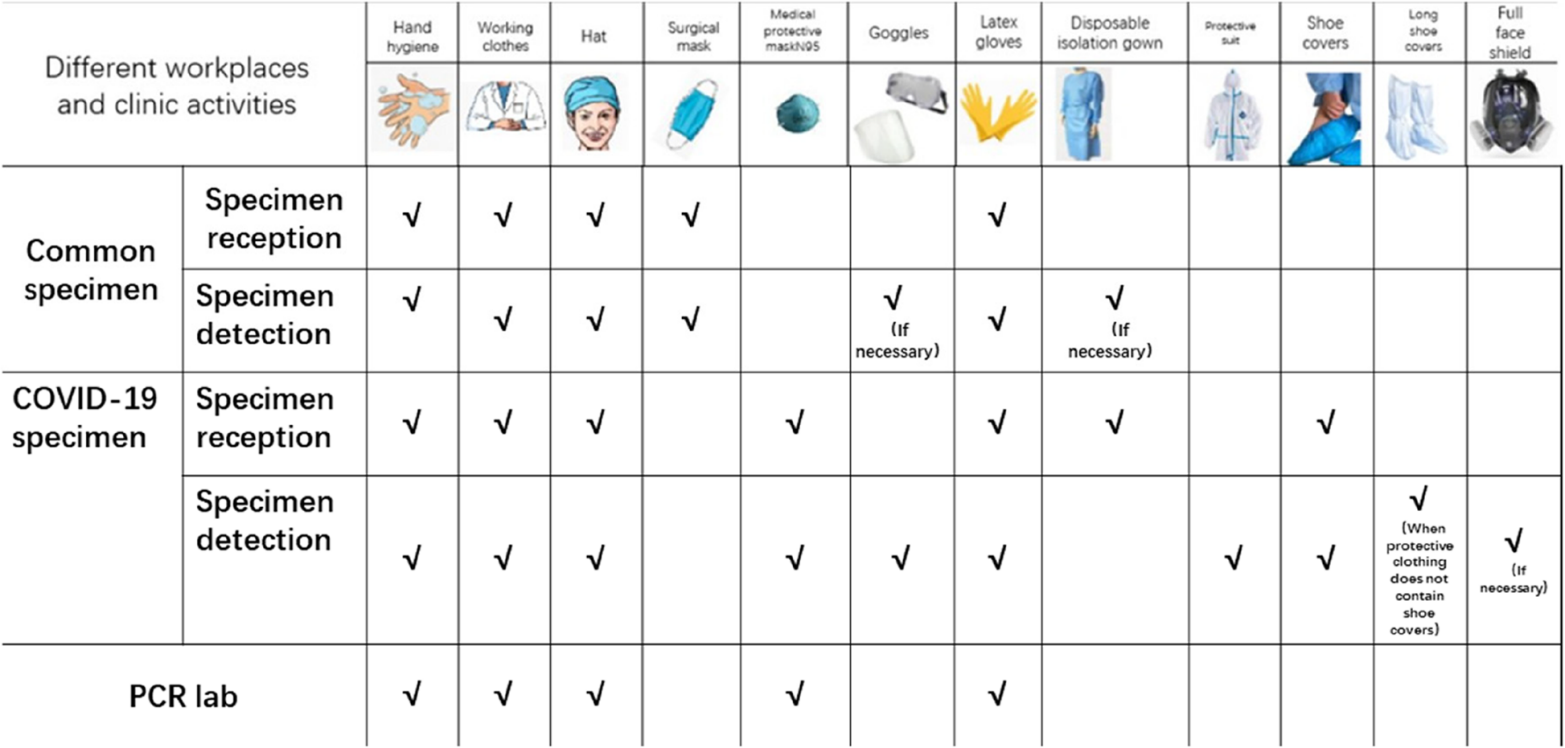
Personal protective equipment requirements in different workplaces

**Fig. 3:**
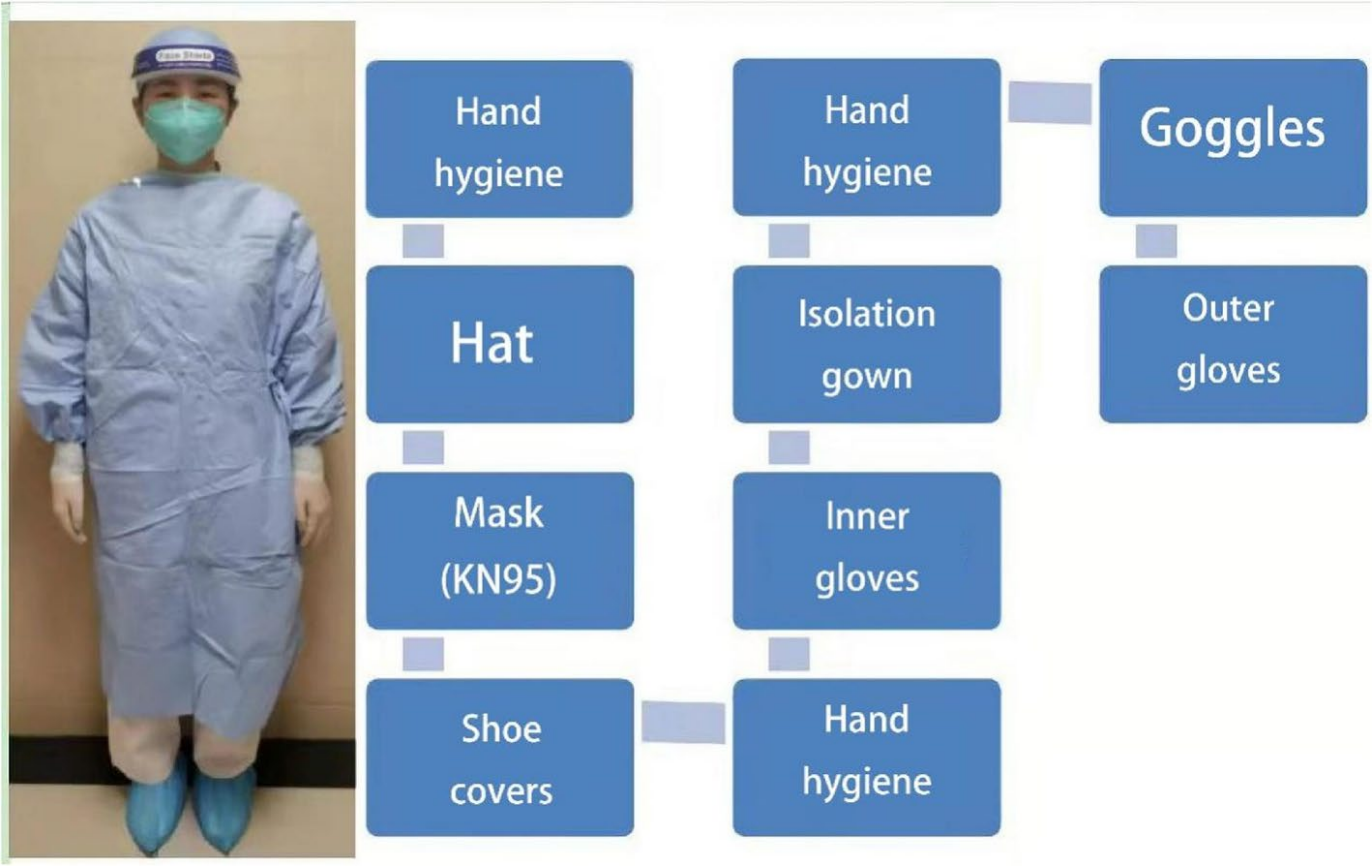
Protection for COVID-19 nucleic acid testing

**Fig. 4:**
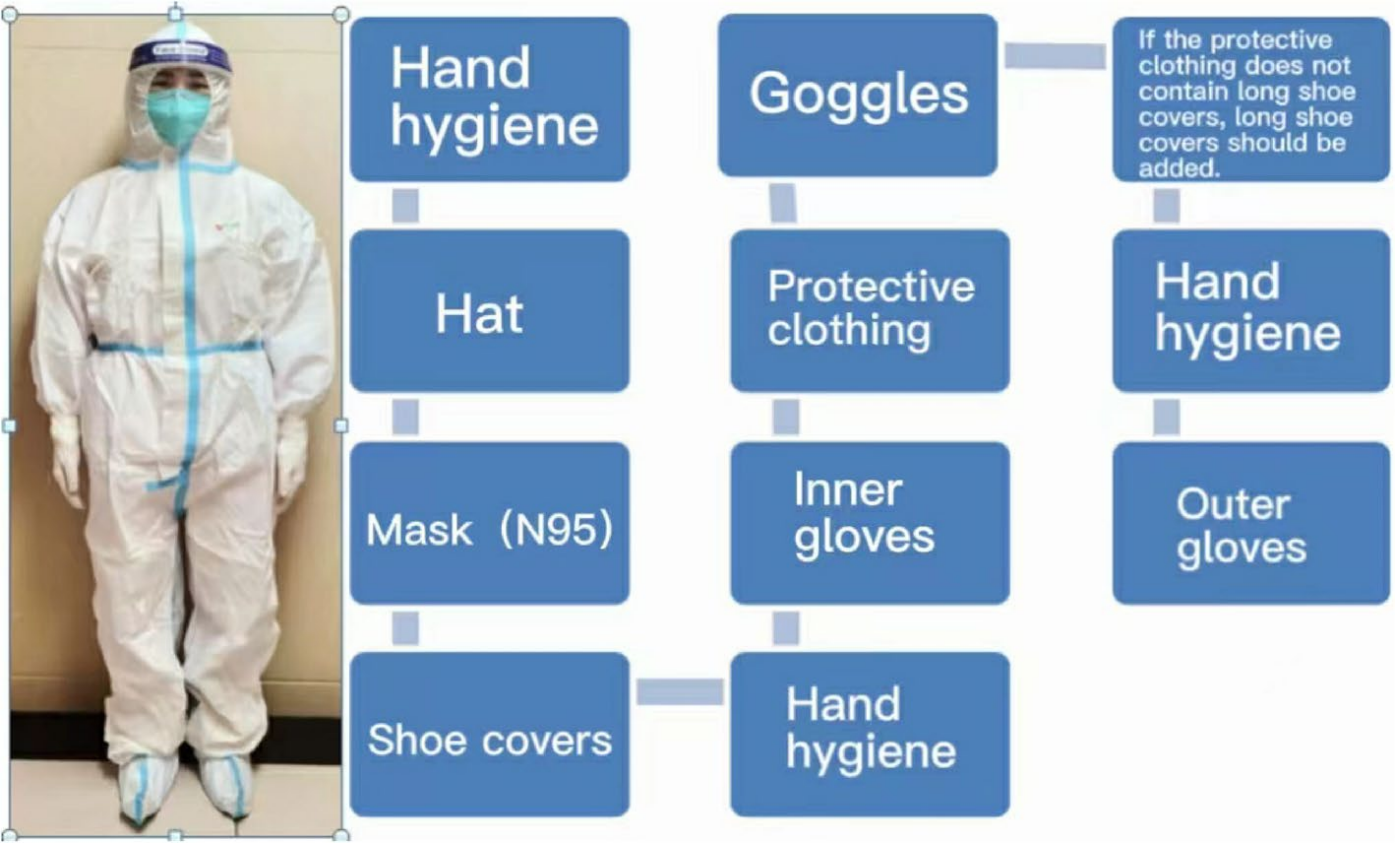
Protection for COVID-19 nucleic acid sample collection

The developers used 3D Studio Max, Unity 3D and Visual Studio to model and display the real environment of the PCR laboratory. Operators can not only roam in the virtual experimental environment, but also enables medical students rapidly to grasp the requirements and biosafety process. There are different requirements for personal protective equipment for handling different specimens in different workplaces (Fig. 1), which are set up according to the specification (Fig. 2). For COVID-19 nucleic acid testing staff wear isolation clothing for experimental operation, as shown in Fig. 3. For COVID-19 nucleic acid sample collection (the most dangerous process), staff wear protective clothing as shown in Fig. 4.

#### Construction of experimental virtual simulation system: (including thinking training and experimental operation module)

**Fig. 5:**
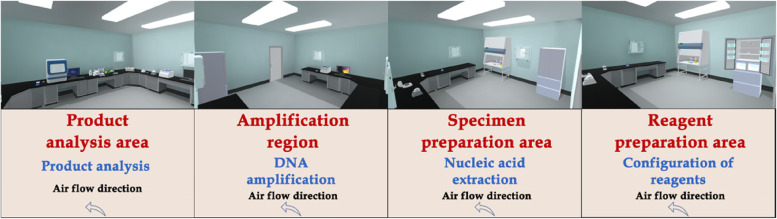
Partition of a gene amplification laboratory

**Fig. 6:**
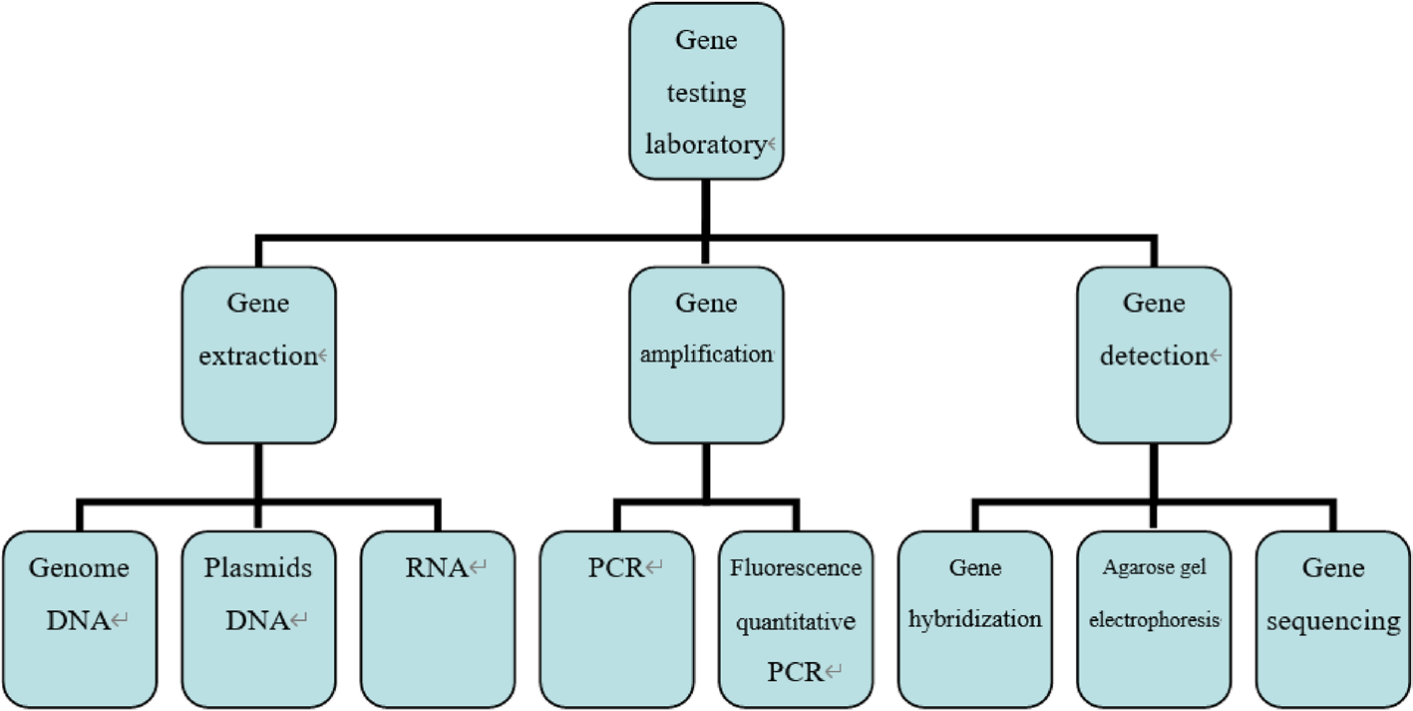
The main functions of genetic testing laboratories

**Fig. 7:**
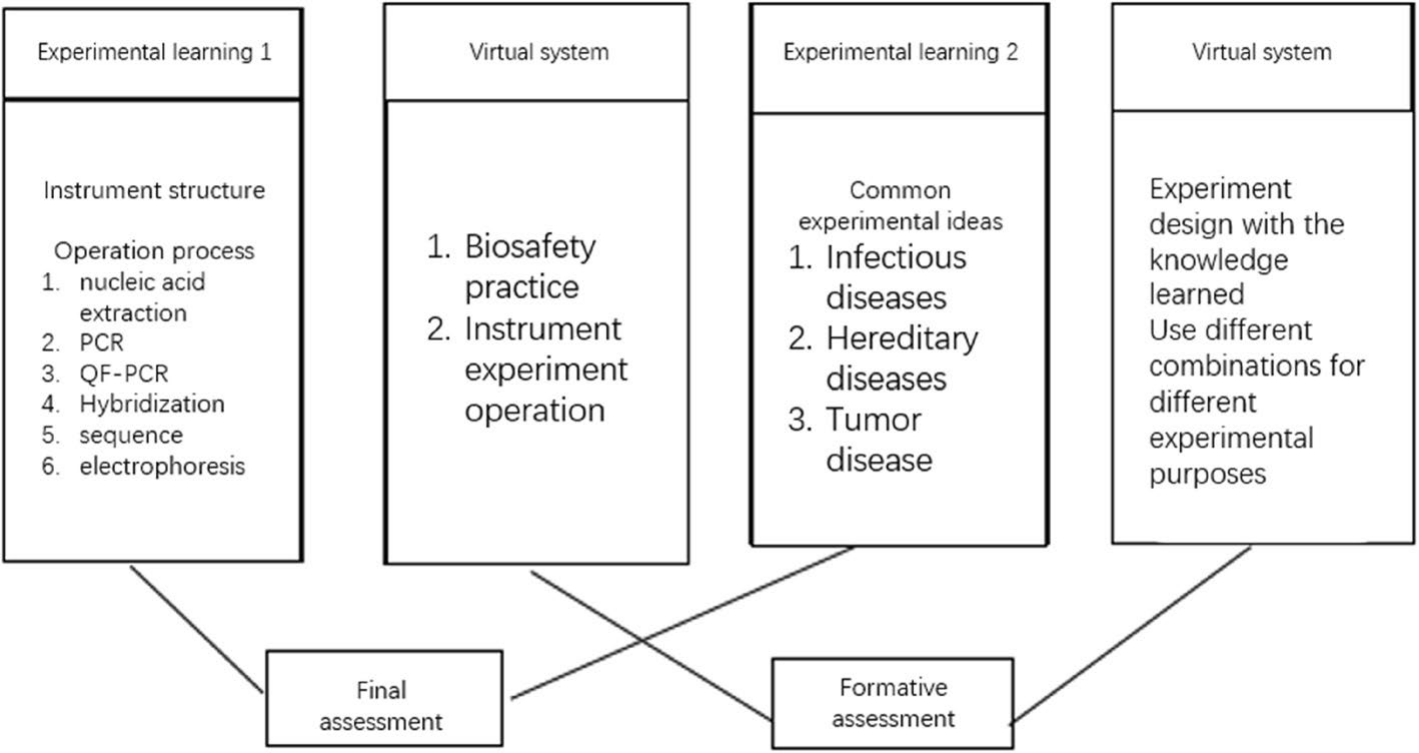
The experimental evaluation system combining summative evaluation and formative evaluation

The system was developed by dividing the laboratory into multiple parts using Unity 3D technology and JAVA. A panoramic view of the laboratory, divided into the following areas: reagent preparation area, sample processing area, PCR area, and product analysis area. The virtual experiment training system was constructed based on the original real materials such as instruments sequencers, biosafety cabinets, centrifuges and operation videos in each area (Fig. 5). The whole system is divided into three parts: gene extraction, gene amplification and gene detection. A comprehensive experimental model/free design combination has been developed in the software (Figs. 6 and 7). Students can choose multiple combinations to complete the experiment to receive training in innovative thinking.

#### Construction of the experiment assessment system: combining summative and formative evaluation

##### Summative assessment

After completing the course, the medical students were assessed on the theoretical content of knowledge, and wrote the experimental report as part of the assessment Formative evaluation: We recorded the correctness or error of each step during the experimental procedure.  Step-by-step and summary scores are provided through the interactive operation that follows. Summative evaluation and formative evaluation each accounts for 50% of the final score.

#### Analysis of teaching effect

The system was launched after its completion, following piloting with medical students from Guangzhou Medical University. A survey was conducted using a questionnaire to obtain feedback from the students. 30 medical students from the molecular diagnosis course participated in the survey, including 10 graduate students and 20 undergraduate students. The data were collected and analyzed in different areas such as learning interest, clinical experimental thinking, experimental operation, perception of how much it helps the student’s own scientific research, biosafety awareness, opinions on the virtual teaching mode, software operability, software difficulties, etc.

## Research results

**Fig. 8:**
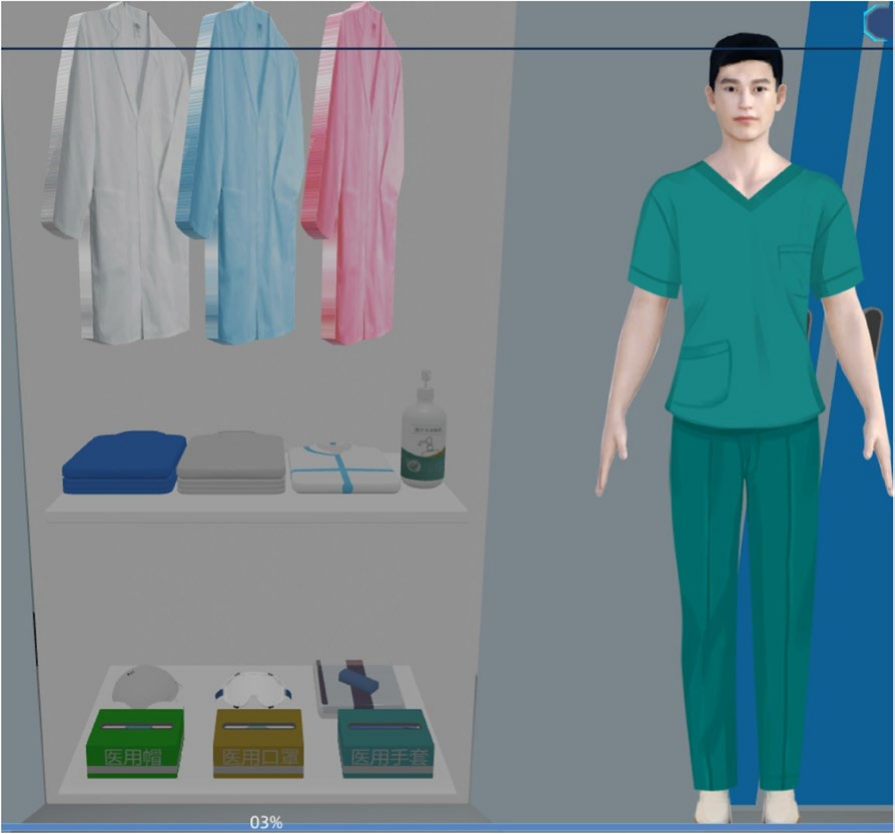
Training for biosafety wear

**Fig. 9:**
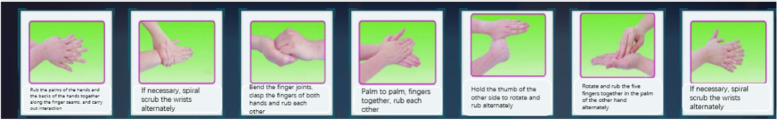
Seven-step handwashing training

**Fig. 10:**
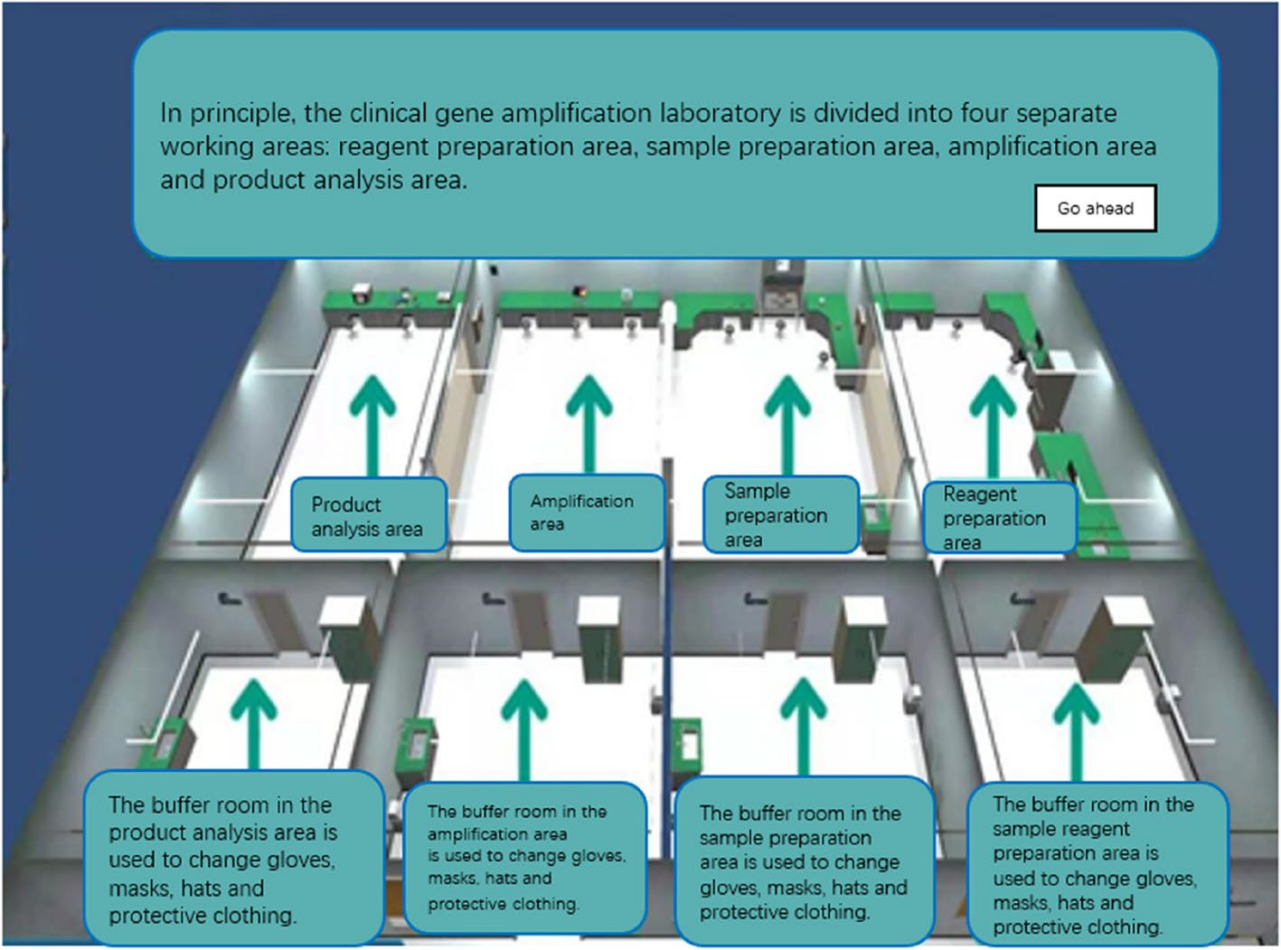
A panoramic view of a PCR laboratory

**Fig. 11:**
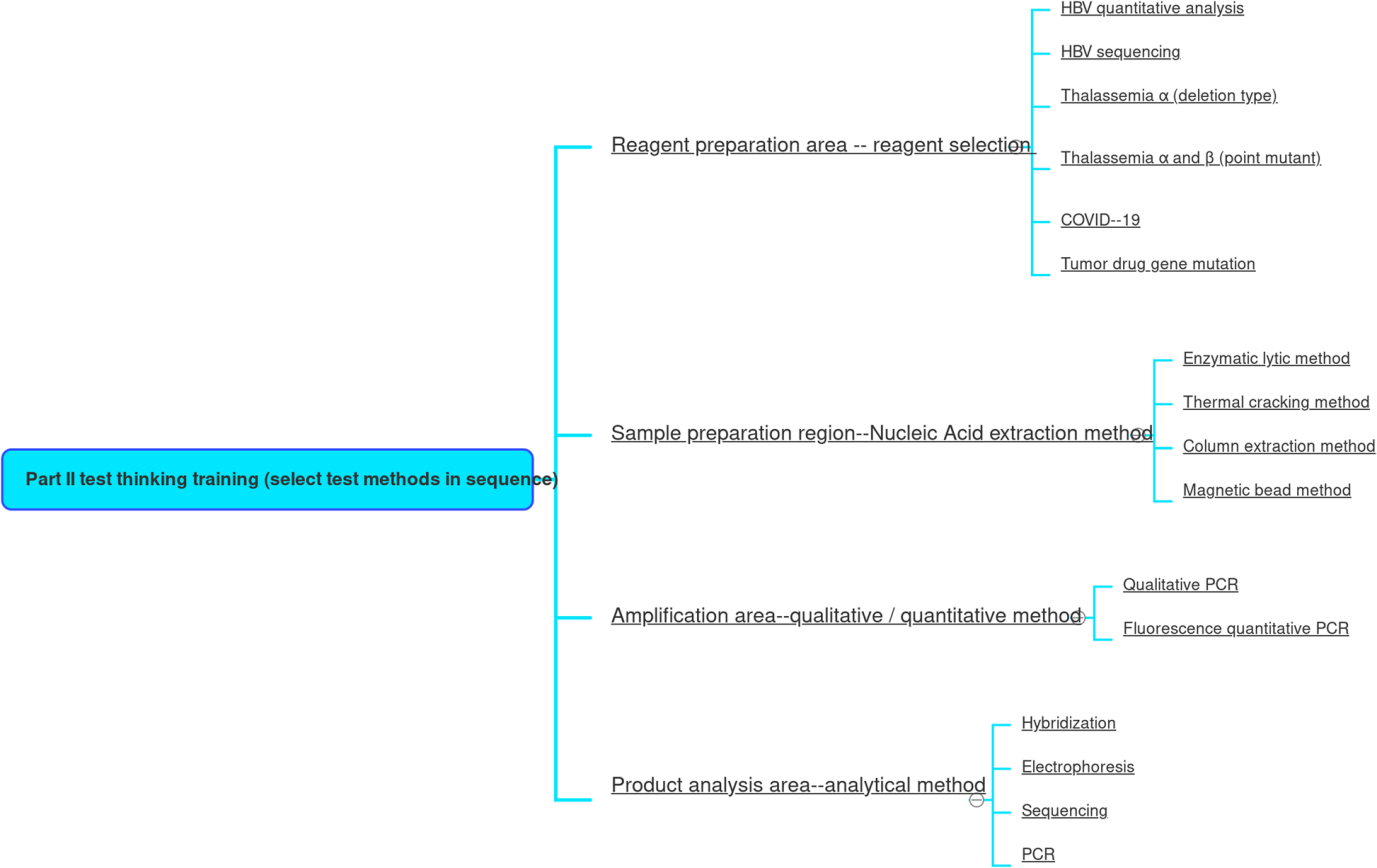
Detection thinking training

1. Successful constructed the laboratory biosafety protection training system, including biosafety protective clothing training (Fig. 8) and seven-step handwashing (Fig. 9).
2. We successfully constructed the virtual simulation system for gene research experiments (including experimental operation and thinking training module). The whole system is divided into four parts: reagent preparation area, gene extraction, gene amplification and gene detection (Fig. 10), and different experimental projects choose different combinations according to the purpose of the experiment (Fig. 11).
3. Successful construction of virtual simulation experiment examination system for medical students.

**Fig. 12:**
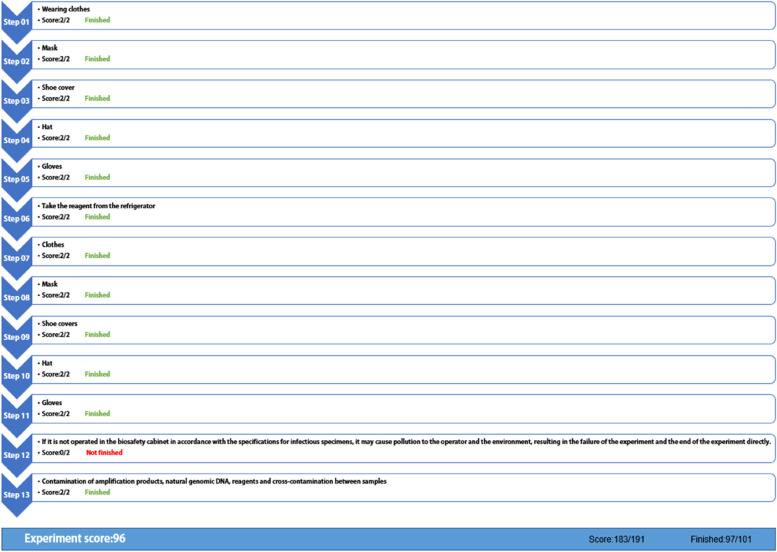
Assessment results for the operation process

**Fig. 13:**
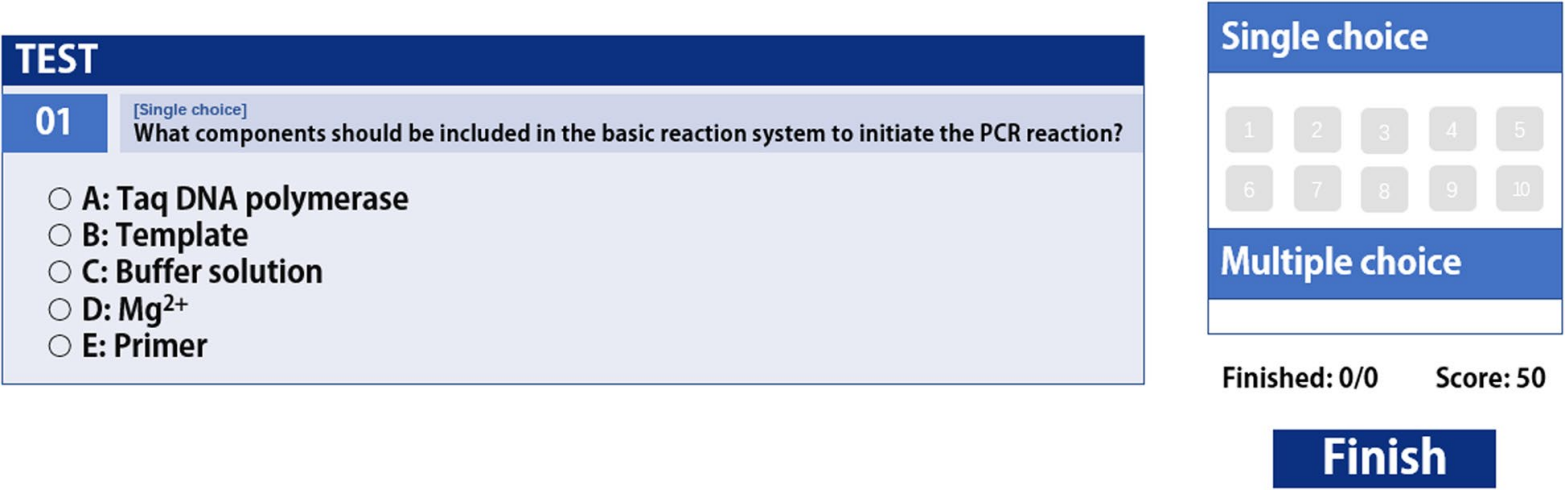
Thinking about the test question

A combination evaluation system of formative assessment and summative assessment was created (Fig. 6). Formative evaluation: teacher’s assessment and evaluation of students can include students’ experimental operations, errors in each step, and links requiring repeated training. The assessment results are fed back to each medical student (Fig. 12), so that they can focus on the key points and difficulties of the experimental operation and avoid the same mistakes in the subsequent real experiments. Concluding evaluation: Students need to complete the experimental report and thinking test questions after the completion of the experimental process (Fig. 13); the score and correct answer will be given after the thinking test questions are completed, and the difficult questions will be given timely feedback.

- 4. Application and evaluation

**Table 1:** Questionnaire of the virtual simulation experiment teaching

Evaluation items	A	B	C	D
Learning interest	Very interested (10)	Quite interested (19)	General interested (1)	No interest (0)
Training of thinking	Very effective (6)	Quite effective (16)	General effective (8)	Not effective (0)
Understanding of PCR experiment	Very sufficient (3)	Quite sufficient (20)	General sufficient (7)	Not sufficient (0)
Help to scientific research	Very helpful (9)	Quite helpful (18)	General helpful (3)	No help (0)
Biosafety awareness	Very strong (10)	Quite strong (18)	General strong (2)	No need (0)
Virtual teaching mode	Very satisfied (8)	Quite satisfied (18)	General satisfied (4)	Not satisfied (0)
Software operability	Very smooth (8)	Quite smooth (21)	Not smooth (1)	Difficult to operate (0)
Software difficulty	Very hard (0)	Quite hard (3)	General (25)	Easy (2)

After training with this virtual software, medical students gave a high evaluation of the teaching activities: 97% of medical students reported that their interest in learning was significantly improved, 85% of the students had been trained in clinical experimental thinking, and 90% of them believed that the help available for scientific research was comprehensive and could meet the needs of later scientific research, allowing them to familiarize themselves quickly with experimental equipment and experimental operations; 93% of medical students believed that it can effectively raise biosafety awareness. Most medical students reported that the software was interactive and guiding, which could improve the ability of independent learning, and this teaching mode was welcomed by 86.7% of students (Table 1).

## Discussion

Medical student education undertakes the task of cultivating medical and scientific research skills [13, 14]. Practical teaching is the key to cultivate medical students in medical colleges. Therefore, improving the quality and effect of practical teaching and improving scientific research achievements are urgent problems to be solved in the teaching of medical students.

Medical students must have biosafety awareness, fully understand and master various experimental skills, and rapidly develop innovative thinking ability to obtain new results. With the increase of the number of medical students in China, teaching work is easily affected by time, funds and space constraints, and the need for practical teaching reform is imminent [15]. The emergence and application of virtual simulation experimental technology provides a new way to solve the above problems. Virtual simulation experimental technology can provide necessary standardization of safe operation, diversification of experimental design, visualization of microscopic experiments and repeatability of experiments that are difficult to carry out, so that students can carry out basic and standardized experimental operation training without entering the laboratory. After mastering the technical principles and experimental operation on line, combined with offline experiments, medical students will soon improve their experimental skills and comprehensive design thinking. The system can also reduce costs, accelerate the research process, improve teaching quality and produce more innovative results [16].

The development of virtual simulation teaching is a good supplement to the actual practical teaching of medical students. This project has completed the simulation construction of the following parts.

First, the laboratory biosafety protection training system was constructed. Experimental biosafety is very important, and the laboratory biosafety protection system is mainly taught through interaction and guidance, so that medical students can understand the most basic protective equipment and master hand hygiene operations. In view of the need for appropriate protective equipment for the treatment of different specimens in different experiments, there are requirements for the protection of experimental personnel according to the specific risks, including the type, quantity and order of wearing protective equipment, which cannot be arbitrary. For example, according to the joint prevention and control mechanism document issuance [17, 18] No. 33, the nucleic acid detection of novel coronavirus requires detection under laboratory conditions of biosafety level II or above to ensure safety. (The universal biosafety level standards are developed by the Centers for Disease Control and Prevention (CDC) and the National Institutes of Health (NIH) in the United States, represented by BSL-1, BSL-2, BSL-3, BSL-4, and P1, P2, P3, and P4, respectively). At present, some related experiments have been arranged for Level III biosafety protection. Students need to be very familiar with Level I, Level II and Level III biosafety protection processes. In addition, standard handwashing is the simplest and most feasible method to prevent cross-contamination. Under the guidance of the system, students can quickly learn and master the standardized hand-washing process by themselves, and at the same time enhance their awareness of hand-washing before and after the experiment through a simple and understandable formula. The design of the PCR laboratory includes a reagent preparation area, a sample preparation area, a amplification area and a product analysis area. In order to avoid cross contamination, we must strictly follow the one-way system to enter each work area, that is, from reagent preparation area to sample preparation area, amplification area to product analysis area.

At the same time, each area should have corresponding laboratory air conditioning and ventilation system design and pressure control to prevent the DNA leakage in the room and diffusion to adjacent areas. Using these knowledge points and through repeated training of virtual software, medical students will gradually become familiar with the process, which is a good way to enhance their biosafety awareness.

Secondly, the virtual simulation system of genetic research experiment is constructed. After entering the laboratory, traditional medical students first learn experimental operation and the use of advanced instruments from senior medical students or technical personnel,, and begin their own research experiments after preliminary mastery. In the absence of standard practices, this learning process can be time-consuming, and learning outcomes can vary from person to person. In the absence of standard practices, this learning process can be time-consuming, and learning outcomes can vary from person to person.. The virtual simulation system has good guidance function: each experimental link is equipped with a navigation bar, and each step of operation is prompted. These courses guide medical students to learn standardized experimental operations in a step-by-step manner [19]. The system can effectively improve the effect of practical teaching and clarify the whole experimental plan and process in a shorter time. For instrument learning, medical students can use virtual simulation experiments to learn, perform instrument operation exercises, and complete various and repeatable practical operations [20]. After repeated training on the virtual simulation platform, medical students showed their accelerated learning of high-tech instrument operation.

Third, the software incorporates a thinking training system for genetic testing. Medical students can be innovatively trained according to the actual situation of learning. They can flexibly combine various methods and instruments in the system to set up experimental content according to different experimental purposes, so as to cultivate experimental thinking. For example, before entering the genetic laboratory, medical students should choose: different genes (from bacteria, viruses, fungi, cells, tissues, etc.) according to the content of the experiment; extraction methods (column extraction, enzyme cleavage, thermal lysis, magnetic bead method); different PCR methods (qualitative, fluorescence quantification, multiplex, amplification refractory mutation system, etc.); different product analysis methods (hybridization, electrophoresis, sequencing, etc.). In this way students can generate different experiment and detection methods to achieve the best results. When designing the experiment, students should choose the corresponding icons on the virtual software according to the experiment purpose so as to select the combined experimental process, so that the students have a very clear idea of the steps before the experiment, and the goal is very clear and it is convenient for each step to carry out the experimental operation correctly. Such training in thinking improves the comprehensive knowledge of medical students and meets national requirements for “innovative, high level and challenging” virtual software. This is where the highlight of this virtual software lies.

Fourthly, we have established an evaluation system for the project. In formative assessment, the virtual software accurately records the situation of each step of the operation, and provides statistical data of scores, main error steps and knowledge points, so that students can immediately understand their learning situation, check defects and fill in gaps. Formative assessment is convenient, especially in the process of biosafety, it seems to be very beneficial when using instruments or manual operation. In addition, in the multi-level and multi-dimensional evaluation model, various technical and disease theoretical knowledge points can be evaluated through summary evaluation, so that students can combine theory with practice, thus improving the quality of experimental teaching [21].

In summary, This virtual simulation experiment has many advantages, but it needs to be clarified that it cannot replace actual clinical operations. Through virtual experiment teaching, medical students are familiar with laboratory methods in advance and undergo repeated training. Therefore, this learning method can greatly improve students' confidence in practice and the standardization of operation. Simultaneously, medical students' awareness of innovation and biosafety has been significantly enhanced efficiently.

It is worth noting that the virtual teaching system needs certain configuration and good network environment. Students’ proficiency in using computer system will also affect their experience of software systems [22, 23].

## Data Availability

All data generated or analysed during this study are included in this published article.
